# Endoscopic submucosal dissection for early gastric cancer using a novel bending attachment

**DOI:** 10.1055/a-2663-2532

**Published:** 2025-08-08

**Authors:** Tomoya Ueda, Takahiro Utsumi, Natsuko Yamahigashi, Yukari Tanaka, Nobukazu Agatsuma, Yuki Nakanishi, Hiroshi Seno

**Affiliations:** 1 Department of Gastroenterology and Hepatology, Kyoto University Graduate School of Medicine, Kyoto, Japan; 2 34797 Department of Medical Equipment, Kyoto University Hospital, Kyoto, Japan

Multibending endoscopes facilitate access to lesions and allow various approaches from different angles during gastric endoscopic submucosal dissection (ESD) 1 2 3 4 5 . However, multibending endoscopes are not widely used owing to their limited purpose and relatively high cost. Furthermore, currently available multibending endoscopes are heavier than standard therapeutic endoscopes, which can place a considerable burden on the operator during long procedures. Herein, we report a case in which gastric ESD was performed using a novel, lightweight, single-use bending attachment (AttachBend; Fujifilm, Tokyo, Japan), which adds a second bending function to a thin therapeutic endoscope (EG-840TP; Fujifilm) ( Fig. 1 ).

**Fig. 1:**
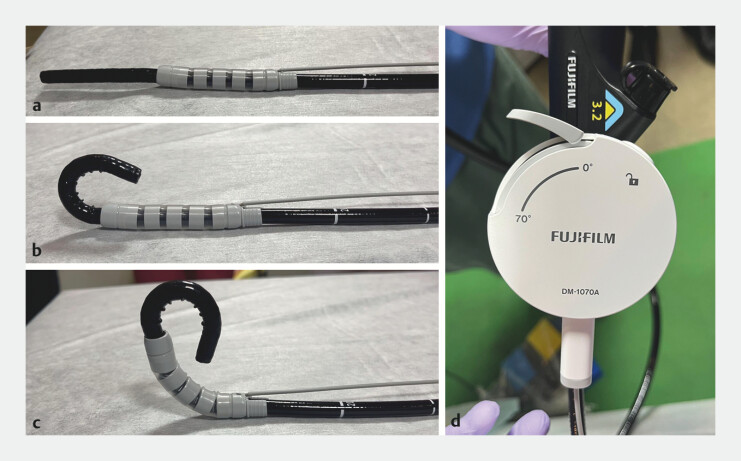
The AttachBend: a novel, lightweight, single-use bending attachment that adds a second bending function to a thin therapeutic endoscope (EG-840TP). **a** The AttachBend is attached to the endoscope. **b** Endoscopic bending at maximum upward angle of endoscope alone. **c** Endoscopic bending at maximum upward angle of endoscope and AttachBend. **d** The angle of the second bending can be adjusted by the operator’s hand.

A 59-year-old man was referred for management of a 15-mm early gastric cancer located in the lesser curvature of the upper body ( Fig. 2 ). ESD was performed using an EG-840TP and an ITknife2 (Olympus, Tokyo, Japan) ( Video 1 ). After the circumferential incision, the submucosa was dissected from the anal side. However, as the submucosal dissection progressed, the muscle layer faced the endoscope, making it difficult to continue efficient and safe dissection while keeping a clear view of the submucosa ( Fig. 3 **a** ). Therefore, an AttachBend was mounted on the endoscope. Its use facilitated the approach to the submucosa parallel to the muscle layer and allowed effective countertraction by insertion of the endoscopic hood between the mucosa and muscle layer ( Fig. 3 **b** ). Additionally, with the addition of a second bend, the knife could be applied to the edge of the submucosa, enabling efficient submucosal dissection ( Fig. 4 ). En bloc resection was successfully achieved without adverse events ( Fig. 5 ). Pathological examination of the resected specimen revealed an intramucosal well-differentiated tubular adenocarcinoma with negative margins.

**Fig. 2:**
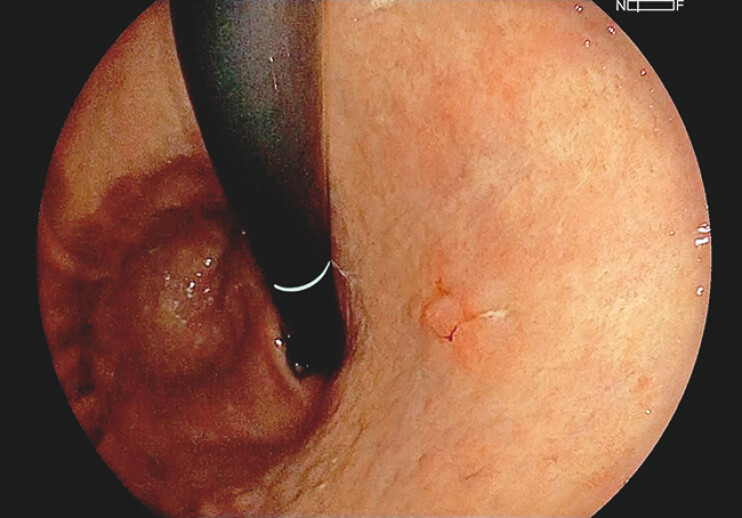
Endoscopic submucosal dissection was performed for a 15-mm early gastric cancer located in the lesser curvature of the upper body.

**Fig. 3:**
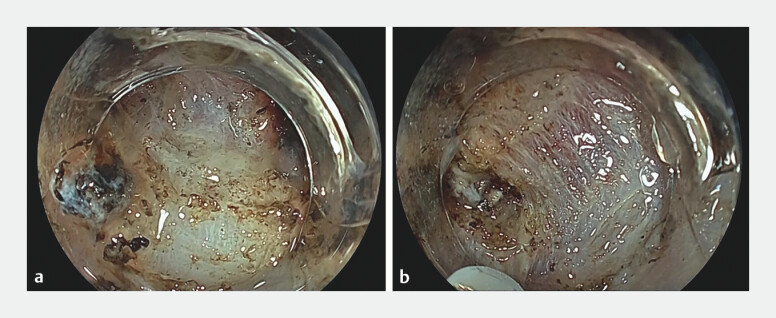
**a** The muscle layer faced the endoscope, which made it difficult to continue efficient and safe dissection. **b** The AttachBend facilitated the approach to the submucosa parallel to the muscle layer and allowed effective countertraction by insertion of the endoscopic hood between the mucosa and muscle layer.

**Fig. 4:**
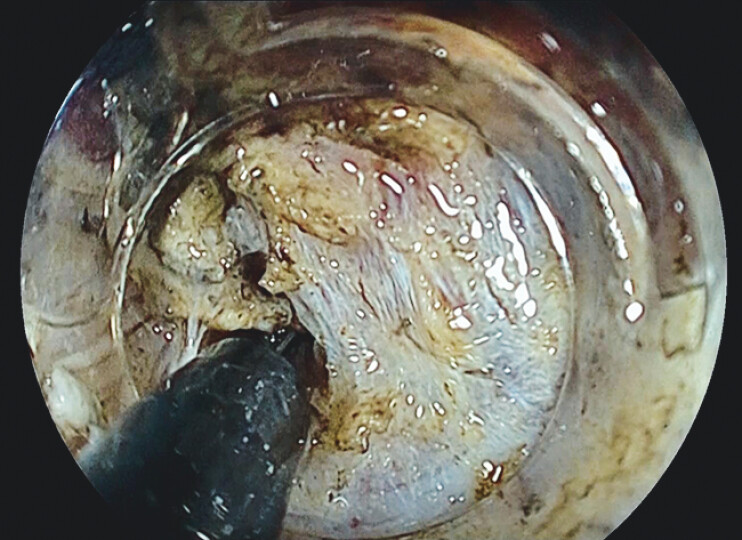
With the addition of the second bend, the knife could be applied to the edge of the submucosa, enabling efficient submucosal dissection.

**Fig. 5:**
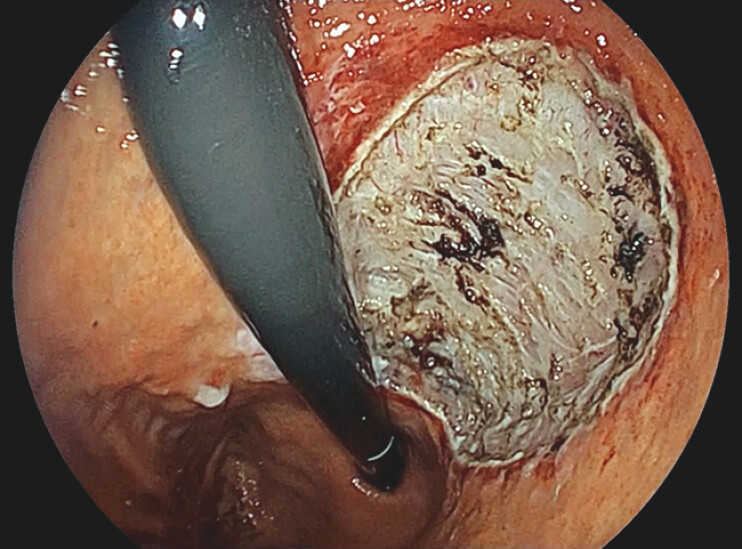
En bloc resection was successfully achieved without adverse events.

Endoscopic submucosal dissection of early gastric cancer using a novel bending attachment, the AttachBend.Video 1

In conclusion, this novel bending attachment would help overcome the difficulties encountered in gastric ESD, such as limited access to the lesion or suboptimal positioning of the endoscope facing the muscle layer, even in the absence of a multibending endoscope.

Endoscopy_UCTN_Code_TTT_1AO_2AG_3AD

## Notes

Endoscopy E-Videos https://eref.thieme.de/e-videos
*E-Videos* is an open access online section of the journal *Endoscopy* , reporting on interesting cases and new techniques in gastroenterological endoscopy. All papers include a high-quality video and are published with a Creative Commons CC-BY license. Endoscopy E-Videos qualify for HINARI discounts and waivers and eligibility is automatically checked during the submission process. We grant 100% waivers to articles whose corresponding authors are based in Group A countries and 50% waivers to those who are based in Group B countries as classified by Research4Life (see: https://www.research4life.org/access/eligibility/ ). This section has its own submission website at https://mc.manuscriptcentral.com/e-videos .
